# Flexible Representations of Dynamics Are Used in Object Manipulation

**DOI:** 10.1016/j.cub.2008.04.061

**Published:** 2008-05-20

**Authors:** Alaa A. Ahmed, Daniel M. Wolpert, J. Randall Flanagan

**Affiliations:** 1 Computational and Biological Learning Lab, Department of Engineering, University of Cambridge, Cambridge CB2 1PZ, United Kingdom; 2 Department of Psychology and Centre for Neuroscience Studies, Queen's University, Kingston, Ontario K7L 3N6, Canada

**Keywords:** SYSNEURO

## Abstract

To manipulate an object skillfully, the brain must learn its dynamics, specifying the mapping between applied force and motion. A fundamental issue in sensorimotor control is whether such dynamics are represented in an extrinsic frame of reference tied to the object or an intrinsic frame of reference linked to the arm. Although previous studies have suggested that objects are represented in arm-centered coordinates [1–6], all of these studies have used objects with unusual and complex dynamics. Thus, it is not known how objects with natural dynamics are represented. Here we show that objects with simple (or familiar) dynamics and those with complex (or unfamiliar) dynamics are represented in object- and arm-centered coordinates, respectively. We also show that objects with simple dynamics are represented with an intermediate coordinate frame when vision of the object is removed. These results indicate that object dynamics can be flexibly represented in different coordinate frames by the brain. We suggest that with experience, the representation of the dynamics of a manipulated object may shift from a coordinate frame tied to the arm toward one that is linked to the object. The additional complexity required to represent dynamics in object-centered coordinates would be economical for familiar objects because such a representation allows object use regardless of the orientation of the object in hand.

## Results

**Figure 1:**
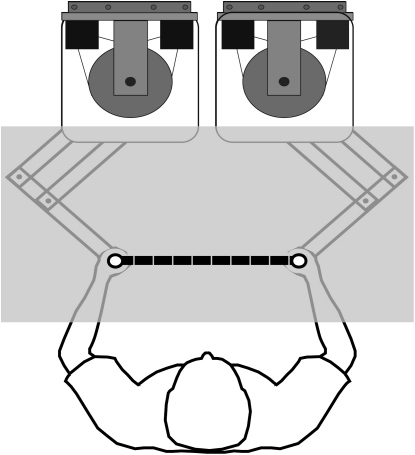
Apparatus While seated, participants grasped two handles, each attached to a planar, force-generating, two-joint, robotic manipulandum. The arms were supported by low-friction air sleds (not shown), which restricted arm motion to the horizontal plane. Participants looked down onto a horizontal semisilvered mirror, located above the hands, that displayed circles representing their hand positions in the plane of arm movement. In the straight-visible condition, participants also viewed an elastic band directly attached to the two handles (as shown in the figure), and in the pulley condition, they viewed an elastic band wrapped around a visible, rotating pulley (see Figure 2).

We used a bimanual object-manipulation task in which participants grasped two handles attached by a virtual elastic band (Figure 1) and moved the right hand to stretch the band while holding the left hand still. In this task, participants learn to compensate for the effects of right-hand movement by generating appropriate forces with the left hand [5, 7–9]. To test whether participants represent dynamics in object- or arm-centered coordinates, we examined how this learning transferred when the object was moved to a new location involving a change in arm configuration.

**Figure 2:**
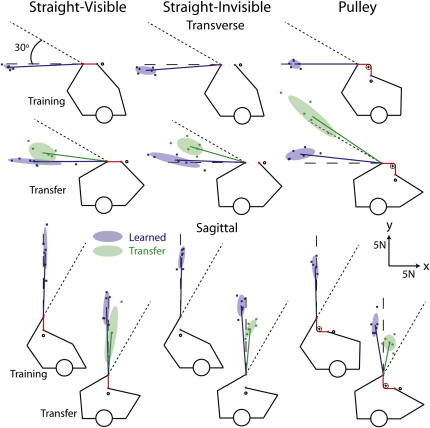
Force Vectors The cartoons depict the arm configurations in the training and transfer positions for all six combinations of object condition (columns) and arm configuration (top and bottom panels). Participants stretched an elastic band (red lines) to a nearby target (black circles) with their right hand. Each blue cross represents the median force vector generated by a given participant during catch trials after learning. The thick blue lines show mean force vectors, averaged across participants, and the blue ellipses represent the corresponding 50% confidence ellipses. Each green cross represents the median force vector during transfer trials after learning in the training position. The thick green lines show mean force vectors, averaged across participants, and the green ellipses represent the corresponding 50% confidence ellipses. The predicted force-vector directions based on transfer in object- and arm-centered coordinates are represented by the dashed and dotted lines, respectively.

Six different groups of participants experienced one of three object conditions in one of two arm configurations (Figure 2). In the straight-visible condition, the elastic band was visible and directly linked between the hands to create an object with relatively simple dynamics. The straight-invisible condition was the same except that the band was not visible. In the pulley condition, the visible band was wrapped around a virtual pulley to create an object with more complex dynamics. Therefore, in the pulley condition the experienced force at the left hand is not parallel to the movement direction of the right hand, whereas, in the straight conditions, the experienced force is parallel to the movement direction of the right hand. Each of these conditions was tested in two arm configurations with the end of the band attached to the left hand oriented in either the transverse or sagittal plane.

The experiment consisted of two phases. In the first phase, participants started with the object in the training position, with the left hand aligned in the sagittal plane with the left shoulder and the left elbow angle at 115°. In standard trials, they were instructed to briefly extend the band 5 cm by moving their right hand to a visible target (black circles in Figure 2) and back while keeping their left hand still. The stiffness of the band was 3.5 N/cm, and therefore the peak force on the left hand was approximately 17.5 N. To assess learning at the training position, we included catch trials (random one in ten) in which we measured the force applied by the left hand while locking the left handle in place. To determine the coordinate frame in which dynamics are represented, we also included trials (one in ten) in which the object was translated to the right (transfer position) such that the left hand was rotated 30° about the shoulder. In these transfer trials, as in catch trials, we measured the force applied by the left hand while locking the left handle in place. After 380 trials (or 38 batches of ten trials), the object was moved to the transfer position and the second phase began. Participants performed an additional 110 trials (11 batches) including catch trials (random one in ten) in addition to the standard trials.

We expected that participants would accurately direct their right hand to the visual target, and would generate similar forces with the right hand, in all conditions and trial types. To test whether this was the case, we computed, for each participant, the median right-hand movement direction, relative to the target, at the time of peak right-hand force as well as the peak right-hand force in five types of trials: standard and catch trials in the training position during phase 1, catch trials in the transfer position during phase 1, and standard and catch trials in the transfer position during phase 2. We then computed, for each arm configuration (two), object condition (three), and trial type (five), averages based on participant medians. Average right-hand direction ranged from −1.3° to 3.4° across the 30 cases, and the average peak right-hand force ranged from 15.4 to 19.9 N. The average time to peak right-hand force ranged from 216 to 339 ms after movement onset. Three-way (two arm configurations × three object conditions × five trial types) ANOVAs revealed a significant effect of trial type on peak right-hand force (F_4,20_ = 4.44; p = 0.02). However, the range of forces across trial types was less than 1 N. No effects of arm configuration, object condition, or trial type were observed for right-hand movement direction. Thus, right-hand movements were consistently directed to the visual target, and similar forces were generated by the right hand in all situations.

**Figure 3:**
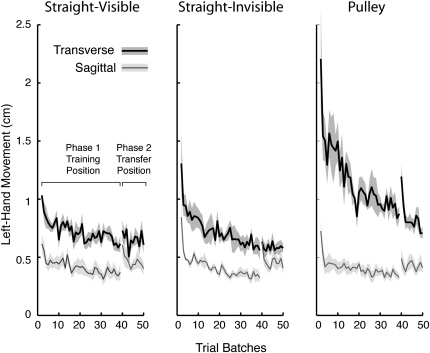
Left-Hand Movement The lines represent mean peak left-hand displacement in the training and transfer positions, averaged across participants, as a function of trial batch. The shaded areas depict ± 1 standard error (SE). Each participant's score is based on the median of the standard trials per batch. Transverse and sagittal arm configurations are shown by thick and thin lines, respectively.

To examine learning in the training position as well as transfer of learning in the transfer position, we computed, for each participant, the median peak displacement of the left hand in each successive batch of standard trials in phases 1 and 2. Figure 3 shows the mean left-hand displacement, averaged across participants, as a function of batch for each object condition and arm configuration. As illustrated in the figure, at the start of phase 1 larger peak displacements were observed in the transverse compared to the sagittal arm configuration. In addition, for the transverse configuration, the peak displacement in the first batch was greatest for the pulley and least for the visible straight band. Paired one-tailed t tests revealed that, for all six combinations of object condition by arm configuration, the peak left-hand displacement in the last batch of ten trials in the training position was significantly smaller than in the first batch of trials (p < 0.05 in all six cases; Bonferroni correction). Thus, significant learning was observed even when manipulating objects with relatively simple dynamics. As can be appreciated in Figure 3, peak left-hand displacement tended to increase slightly when the object was translated to the transfer position at the start of phase 1 but was generally smaller than at the beginning of phase 1.

To quantify steady-state performance in the training position, we computed, for each participant, the median force vector generated by the left hand over the last 19 catch trials (i.e., the last half) delivered at the training position. To assess transfer, we computed the median force vectors over the last 19 transfer trials. Finally, to quantify steady-state performance after learning at the transfer position, we computed the median force vector over the last six catch trials delivered at the transfer position. The blue lines in Figure 2 show the force vectors for the catch trials in the training and transfer positions. These lines represent left-hand performance at these two positions after learning. The green lines show the force vectors for the transfer trials and represent the generalization of learning from the training to the transfer position before the left hand experienced forces in the transfer position. Note that the average peak force generated by the left hand in catch and transfer trials (11.4 N) was smaller than the average force (16.9 N) generated by the left hand in standard trials. This indicates that only a component of the robot-generated force on the left hand, in standard trials, is compensated for with predictive force generation. The remaining force in standard trials is presumably counteracted with a combination of mechanical stiffness (because the left hand is slightly perturbed) and reflexive increases in force.

If object dynamics are represented in arm-centered coordinates, we would expect the force vector generated by the left hand in transfer trials to rotate with the arm and thus be rotated 30° (dotted lines in Figure 2) from the direction of the elastic force. Conversely, if object dynamics are represented in object-centered coordinates, we would expect the force vector to be aligned with the direction of the elastic force (dashed lines in Figure 2). As illustrated in Figure 2, when vision was available the dynamics of the straight band were primarily represented in object-centered coordinates, whereas the dynamics of the band and pulley were primarily represented in arm-centered coordinates. Moreover, when vision of the straight band was removed, an intermediate representation was observed.

**Figure 4:**
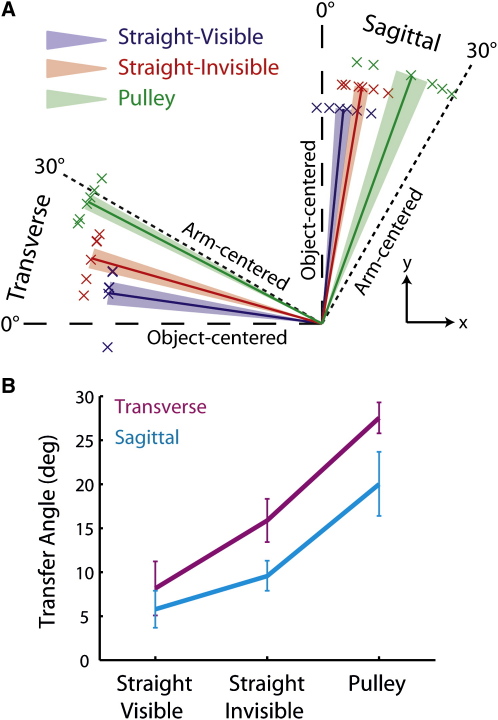
Transfer Angles (A) The transfer angles for all object conditions and arm configurations are presented as normalized vectors in polar coordinates. For clarity, the vectors for the object conditions are scaled differently. Each cross represents the median transfer angle for a single participant, and the thick lines show mean angles averaged across participants. The shaded areas represent ± 1 SE. Object-centered and arm-centered predictions are represented by dashed and dotted lines, respectively. (B) The magnitude of the average transfer angle (±1 SE) for each object-condition is presented. Transverse and sagittal arm configurations are shown in purple and cyan, respectively.

To quantify transfer of learning, we evaluated transfer performance relative to steady-state performance at the transfer position. That is, for each participant, we subtracted the angle of the median force vector in transfer trials from the angle of the median force vector in catch trials delivered after learning at the transfer position. Figure 4A shows the resulting transfer angles for the three object conditions and two arm configurations in polar coordinates on three unit circles. An angle of zero would indicate perfect transfer in object-centered coordinates, and an angle of 30° would indicate perfect transfer in arm-centered coordinates. To assess the effects of condition and configuration on transfer angle, we carried out a two-way (three by two) between-subjects ANOVA. Significant effects of both object condition (F_2,30_ = 22.5; p < 0.001) and arm configuration (F_1,30_ = 6.7; p = 0.015) were observed, but there was no interaction (F_2,30_ = 0.55; p = 0.58). Figure 4B shows the means and standard errors for each combination of object condition by arm configuration. The effect of object condition was assessed further with two planned comparisons collapsing across arm configurations. To determine the effect of dynamics complexity, we compared the combination of the two straight-band conditions against the pulley condition. The transfer angle was greater in the pulley condition (p < 0.001). To assess the role of vision of the object, we compared the two straight-band conditions. The transfer angle was greater when the object was not visible (p = 0.043). In addition to these planned comparisons, we carried out post-hoc comparisons, by using a Bonferroni correction, to determine whether the transfer angle in the straight-visible and pulley conditions was significantly different than 0° and 30°, respectively. Collapsing across arm configurations, we found that the transfer angle in the straight-visible condition (M = 6.9°) was reliably greater than 0° (p = 0.006) and that the transfer angle in the pulley condition (M = 23.7°) was reliably less than 30° (p = 0.034). Thus, transfer in the straight-visible and pulley conditions was primarily, but not purely, in object- and arm-centered coordinates, respectively.

**Figure 5:**
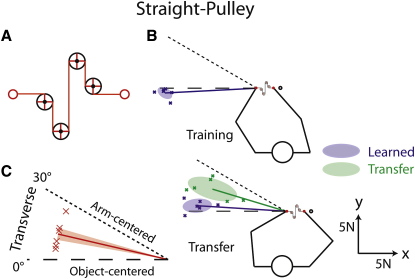
Straight-Pulley Condition (A) Locations of the hands (red circles) and four pulleys used in the straight-pulley condition. (B) Each blue cross represents the median force vector generated by a given participant during catch trials after learning. The thick blue lines show mean force vectors, averaged across participants, and the blue ellipses represent the corresponding 50% confidence ellipses. Each green cross represents the median force vector during transfer trials after learning in the training position. The thick green line shows the mean force vector, averaged across participants, and the green ellipse represents the corresponding 50% confidence ellipse. (C) Each red cross represents the median transfer angle for a single participant, and the thick red line shows mean angles averaged across participants. The shaded areas represent ± 1 SE. Object-centered and arm-centered predictions are represented by dashed and dotted lines, respectively.

It is possible that the difference in generalization between the straight-visible and pulley conditions is related to visual complexity rather than mechanical complexity. Therefore, we ran an additional group of participants by using an object with simple mechanics but high visual complexity. We simulated an elastic band running through a set of four pulleys (Figure 5A). Although visually complex, the mechanics in this straight-pulley condition are identical to those in the transverse straight conditions. The results for this condition are shown in Figures 5B and 5C, which correspond to Figures 2 and 4A, respectively. The blue lines in Figure 5B show the average force vectors generated by the left hand during catch trials delivered (after learning) at the training and transfer positions in phases 1 and 2, respectively. The green line shows the average force vectors during transfer trials delivered at the transfer position in phase 1. Figure 5C shows the average transfer angle—the angle of the force vector in transfer trials (phase 1) minus the angle of the force vector in catch trials delivered at the transfer position (phase 2). The results for the straight-pulley are very similar to those obtained for the straight-invisible band in the transverse configuration (see Figure 2). In particular, transfer was intermediate between object-centered and arm-centered coordinates. Thus, although participants could not exploit the complex visual feedback provided in the straight-pulley condition to form an object-centered representation, the complex visual feedback did not lead to encoding in arm-centered coordinates. In other words, the results suggest that the arm-centered encoding seen in the pulley conditions cannot be explained on the basis of visual complexity alone.

## Discussion

Numerous studies have demonstrated that humans learn and maintain internal models or representations of object dynamics that are used both to estimate the motor commands required to achieve desired outcomes and to predict the sensory consequences of our actions [1, 10–19]. Moreover, neurophysiological studies have shown that nonhuman primates are able to learn the kinematics and dynamics of novel tools [20–23]. However, relatively few studies have examined the coordinate frame in which object dynamics are learned. The results presented here suggest that the way in which we represent object dynamics is flexible and depends on the complexity of the dynamics. When experiencing the elastic band wrapped around a pulley, participants represent the relatively complex dynamics in arm-centered coordinates. The idea that complex dynamics are encoded in arm-centered coordinates agrees with the results of previous studies that have employed objects with unusual and complex dynamics [1–5]. In contrast, our results indicate that objects with simpler dynamics can be represented in object-centered coordinates. After participants learned to minimize left-hand movement when manipulating the straight visible band, this learning generalized in object-centered coordinates.

Although we have argued that objects with simpler dynamics are encoded in object-centered coordinates, our results are also consistent with the idea that such objects are encoded in Cartesian coordinates. However, we favor the object-centered interpretation because such a representation would enable generalization across changes in object orientation. Because we used a virtual environment to simulate various mechanical objects, the question arises of whether similar results would be obtained with real objects (i.e., springs and pulleys). Although we cannot rule out the possibility that differences would be observed, we believe that our objects were far more natural than those used in previous studies because we simulated objects with natural physics. In addition, the fact that the dynamics of one of our objects (the straight-visible spring) were encoded in object-centered coordinates suggests that our simulations were effective.

We found that when the straight elastic band was not visible, dynamics were learned in an intermediate frame of reference between arm- and object-centered coordinates. This finding indicates that the representation of dynamics is not categorical—that is, either arm- or object-centered. Indeed, some mixture of coordinate frames was evident even in the straight-visible and pulley conditions. This result also suggests that vision of an object can facilitate encoding of dynamics in object-centered coordinates. Note that, in addition to using objects with complex dynamics, most previous studies demonstrating that dynamics are learned in arm-centered coordinates have not provided visual cues of dynamics [1–5]. We did not include an invisible pulley condition because pilot data indicated that transfer occurred in arm-centered coordinates and arm-centered encoding was observed even when vision of the pulley and spring was provided. Using the same apparatus and general approach employed in the present study, a previous study [5] examined an object with complex dynamics (where the force applied to the left hand depended on the velocity of the right hand but acted at right angles to right-hand movement direction) without visual cues about dynamics. Their observation that dynamics were encoded in pure arm-centered coordinates supports our conclusions that both dynamics complexity and visual cues about dynamics can influence the way in which object dynamics are represented.

Although we have suggested that the way in which people encode object dynamics depends on the complexity of dynamics, the critical factor influencing how dynamics are encoded may be experience or familiarity. Thus, if an individual frequently experienced pulleys, we would expect him or her to represent our visible pulley in object-centered coordinates. We suggest that with experience with a particular class of objects, the representation of dynamics shifts from an arm-centered coordinate frame to an object-centered coordinate frame. An arm-centered frame may be developed initially as we experience an object in particular configurations relative to our hands and arms. However, as we gain experience with different configurations, we may begin to develop a more general object-centered representation that is independent of the position and orientation of the object relative to the hands and arms.

## Experimental Procedures

Forty-two right-handed participants (22 males, 20 females, 18–35 yr) took part in the study after providing written informed consent. The experimental procedures were approved by the Local Ethics Committee.

### Equipment

Seated participants made two-dimensional reaching movements in the horizontal plane while holding the handle of a planar, force-generating, two-joint, robotic manipulandum (vBOT) [24] in each hand (Figure 1). Shoulder straps restrained the upper body, and both arms were supported by low-friction air sleds with the upper and lower arms in the horizontal plane. Participants looked down onto a horizontal mirror that reflected the display of an LCD monitor suspended above. The monitor displayed circles representing the participants' hand positions, targets, and task-relevant visual feedback in the plane of the arm movements. The display was calibrated so that visual feedback of the hands was overlaid on the true hand position.

### Experimental Protocol

Participants were informed that an elastic band would be simulated between their hands and that they were required to stretch one edge of the band to a target with their right hand while keeping their left hand still.

During each trial, start positions (1 cm radii) of the two hands were displayed as well as a target for the right hand (1 cm radius). Cursors (0.5 cm radii) representing each hand position were always visible. A position-dependent force linked the hands, simulating an elastic band with a linear spring constant of 3.5 N/cm. With both hands in the start positions, the band was not in tension and was slack until the band extended by 0.4 cm. A trial consisted of making an out-and-back movement with the right hand to the target while keeping the left hand as still as possible. Once the participant's hands were in their respective start positions, he or she waited for an audio cue to begin the out-and-back movement with the right hand. The target appeared 5 cm away from the right-hand start position. Participants received warnings when movements were too slow (greater than 1 s) or if left-hand movement was greater than 1 cm.

Participants learned the task in one workspace location (training) and were tested for generalization at another location (transfer). During training, the start position for the left hand was aligned with the left shoulder in the sagittal plane, with a 115° elbow angle. The transfer position was obtained by rotating the left arm clockwise 30° about the left shoulder while maintaining a constant elbow angle (Figure 2). The right hand moved such that the object maintained its orientation in Cartesian coordinates.

Participants were randomly allocated to one of six groups (each of six subjects), and each group was exposed to one of three object conditions (straight-visible, straight-invisible, or pulley) in one of two arm configurations (transverse or sagittal) (Figure 2). In the straight conditions, the object was an elastic band connected between the hands, which were 12 cm apart. In the straight-visible condition, a striped band was visible between the two hand positions. A force applied by one hand resulted in a force of equal magnitude and opposite direction upon the other and an appropriate widening of the visible stripes on the band. In the straight-invisible condition, the elastic band was invisible. In the pulley condition, the elastic band was visible and wrapped 90° around a pulley (2 cm radius with four visible spokes), which rotated as the band was stretched. The two hands were both 10 cm from the center of the pulley in orthogonal directions.

An additional six subjects were exposed to an additional object condition in the transverse configuration. In this straight-pulley condition, a visible elastic band was wrapped around four pulleys (1 cm radii, with four visible spokes, Figure 5A), so that both ends of the band were aligned in the sagittal plane. The hand positions and dynamics of this condition were identical to the straight-visible and straight-invisible conditions in the transverse plane.

During standard trials, the hands were dynamically linked by the spring. For the quantification of learning and the measurement of the magnitude and direction of the force anticipated by the left hand, catch trials were included in which the hands were unlinked. The left hand was locked in position by a high-stiffness virtual spring (35 N/cm) while the right hand was allowed to move to the target and experienced the same position-dependent force as in the standard trials.

For familiarizing participants with the task, they performed five trials in the training position and five in the transfer position in which they moved their right hand to the target and the left handle was locked in place. Participants then performed 38 batches of ten trials each. The first nine trials of every batch were in the training position, and one of these trials (other than the first or last) was randomly selected to be catch trial. The last trial of each batch was also a catch trial, but in the transfer position. No forces were experienced and no visual feedback of the object was provided while participants moved between the training and transfer positions. Participants then performed 11 batches in the transfer position. One trial in every batch (other than the first or last) was chosen at random to be a catch trial.

### Data Acquisition and Analysis

The two-dimensional position and robot-generated forces were recorded for each hand at 1000 Hz. For assessment of performance, the direction and magnitude of the force generated by the left hand at the time of maximum force in the right hand was calculated for all catch trials. We also computed peak left-hand displacement, peak right-hand force, time to peak right-hand force, and the direction of right-hand movement at the time of peak right-hand force.
